# Low dose ultraviolet B irradiation at 308 nm with light-emitting diode device effectively increases serum levels of 25(OH)D

**DOI:** 10.1038/s41598-021-82216-1

**Published:** 2021-01-28

**Authors:** Ming-Yen Lin, Lee Moay Lim, Siao-Ping Tsai, Feng-Xuan Jian, Shang-Jyh Hwang, Yu-Hsuan Lin, Yi-Wen Chiu

**Affiliations:** 1 Division of Nephrology, Department of Internal Medicine, Kaohsiung Medical University Hospital, Kaohsiung Medical University, No. 100, TzYou 1st Rd., Sanmin District, Kaohsiung City, 80708 Taiwan; 2 Department of Renal Care, College of Medicine, Kaohsiung Medical University, Kaohsiung, 807 Taiwan; 3 Graduate Institute of Medicine, College of Medicine, Kaohsiung Medical University, Kaohsiung, 807 Taiwan; 4 Institute of Population Health Sciences, National Health Research Institutes, Miaoli, 350 Taiwan; 5 Faculty of Medicine, College of Medicine, Kaohsiung Medical University, Kaohsiung, 807 Taiwan; 6 Taiwan Instrument Research Institute, National Applied Research Laboratories, Hsinchu, 300 Taiwan

**Keywords:** Disease prevention, Nutrition

## Abstract

This animal study aimed to elucidate the relationship of low-dose, narrow-band UVB at 308 nm with vitamin D synthesis. C57BL/6 female mice, at 3 weeks-of-age, were randomly divided into the following six groups (n = 6 at each time point of vitamin D measurement), which were: (1) normal diet without UVB irradiation; (2) VDd diet without UVB irradiation; and (3)–(6) VDd diet with 308 nm-UVB irradiation of 12.5, 25, 50, and 100 μω/cm^2^, respectively. All of the groups needing UVB irradiation received an exposure of 10 min per day, five days per week, and a duration of 3–5 weeks. The mice recovering from severe VDd (plasma total 25-hydroxyvitamin D level increasing from approximately 3 to over 30 ng/mL) only occurred in groups with a UVB irradiation dosage of either 50 or 100 μω/cm^2^. The optimal, estimated dosage for mice to recover from severe VDd was 355 mJ/cm^2^ within 3 weeks. Low-dose, narrow-band UVB irradiation at 308 nm is effective in improving VDd in mice. The results obtained, in addition to the especially small side effects of the above UVB irradiation formula, could be further translated to treating VDd-related disorders.

## Introduction

Vitamin D is crucial in numerous physical functions, and vitamin D deficiency (VDd) may cause major defects in bone mineralization and metabolic function, such as osteoporosis, rickets, muscle weakness, cardiovascular disease, and even immune dysfunction^1–3^. The primary source of vitamin D in humans comes from cutaneous synthesis, in which 80–100% of vitamin D requirement is received through ultraviolet B (UVB) radiation from sunlight^4^.

In human, vitamin D is produced in the epidermis by photochemical transformation, where 7-dehydrocholesterol is subsequently hydroxylated to prduce biologically active 1α,25-dihydroxyvitamin D (1,25(OH)_2_D_3_)^5,6^. In this canonical pathyways, vitamin D is then transported to the liver where it is hydroxylated at C25 by CYP27A1 or CYP2R1 to form 25(OH)D_3_. The 25(OH)D_3_ is then hydroxylated at C1αeither in the kidney or peripheral tissues expressing CYP27B1 to form 1,25(OH_2_)D_3_^7–9^. Slominski A.T. et al. have described a novel noncanonical pathway of vitamin D activation by CYP11A1 to produce a variety of metabolites including 20(OH)D3 and 20,23(OH)_2_D_3_^10–12^. CYP11A1 similarly initiates the metabolism of lumisterol (L3) through sequential hydroxylation of the side chain to produce 20(OH)L_3_, 22(OH)L_3_, 20,22(OH)_2_L_3_ and 24(OH)L_3_. CYP11A1 also acts on 7-dehydrocholesterol (7DHC) producing 22(OH)7DHC, 20,22(OH)27DHC and 7-dehydropregnenolone which can be converted to the vitamin D and L_3_ configurations following exposure to UVB^13^. These CYP11A1-derived compounds are detected in human epidermis and serum, and pig adrenal gland, and are biologically active displaying anti-proliferative, anti-inflammatory, anti-cancer and pro-differentiation properties^14^.

A recent study demonstrated that vitamin D insufficiency is prevalent worldwide, with the rate mainly around 28–97% of the adult population when defined as serum 25-hydroxyvitamin D [25(OH)D] concentrations less than 30 ng/mL^4^. Taiwan is one of the most VDd countries globally, even though it is located in subtropic zones with abundant sunlight. This suggests that factors other than sunlight, such as aging, skin pigmentation, seasonal use of sunscreen, working environment, outdoor physical activities, and air pollution, could contribute to VDd in modern societies^15–19^. Given the relatively low exposure to sufficient UVB from sunlight, irrespective of intentionality, all nutritional guidelines recommend oral supplement of vitamin D^20^. VDd, however, remains ubiquitous worldwide and probably the consequence of unawareness, inaccessibility, and poor compliance^21^.

Considering the general lack of compliance of taking vitamin D supplements and in order to avoid oral vitamin D toxicity due to over-dosage, UV lamps, as a component of home lighting, could constitute a viable alternative to overcome common VDd^22^. Effectiveness of vitamin D synthesis, and adverse effects such as carcinogenesis, erythema injury, and DNA damage are mainly determined by spectrum of UVB^23,24^ that should be carefully considered in developing UV lamps.

UV absorption by the skin triggers the transduction of UV electromagnetic energy into chemical, hormonal, and neural signals, defined by the nature of the chromophores and tissue compartments receiving specific UV wavelength. UV radiation can upregulate local neuroendocrine axes, with UVB being markedly more efficient than UVA^25^. The locally induced cytokines, corticotropin-releasing hormone, urocortins, proopiomelanocortin-peptides, enkephalins etcs, can be released into circulation to exert systemic effects, including activation of the central hypothalamic–pituitary–adrenal axis, opioidogenic effects, and immunosuppression, independent of vitamin D synthesis^25^. Exposure of UVB to the eyes and skin activates hypothalamic paraventricular and arcuate nuclei, and exerts rapid stimulatory effects on the brain resetting body homeostasis^25^. This implicates multiple therapeutic applications of UV radiation in the management of autoimmune and mood disorders, addiction, and obesity.

Although UVB irradiation dosage has been shown to be positively associated with degrees of the above-mentioned adverse effects, UVB wavelength plays a key role in negative biologic effects. For erythema and DNA damage action spectrums from solar, wavelengths of less than 300 nm are more likely to result in molecule structure changes compared with the range between 300 and 320 nm. Moreover, for vitamin D synthesis and carcinogenesis action spectrums, UVB shares a similar wavebands on both biologic effects. Narrow-band rather than broad-band UVB phototherapy has been proven to be more effective in improvement of VDd, but few of these investigations focused on UVB adverse effects^26^. To identify a UVB spectrum aimed to improve VDd safely, we referred to our prior study and chose the UVB band at approximately 308 nm, given its desirable balance of biologic effects between the vitamin D synthesis with other adverse ones^27,28^. Therefore, we carried out this animal study to elucidate the effects of narrow-band UVB at 308 nm and irradiance less than the standard erythema dose (SED) (equivalent to an erythemal effective radiant exposure of 100 J/m^2^) on increasing vitamin D synthesis.

## Results

### Effects of low-dose UVB irradiation on serum 25(OH)D levels

Table 1 presents the effects of 308 nm UVB irradiation on serum 25(OH)D levels according to dose. The normal diet group maintained 25(OH)D levels at approximately 30 ng/ml during the whole experiment, although a slight decrease occurred at the conclusion of the study. As anticipated, the VDd group’s mean vitamin D levels were close to the lower limit of detection, 3 ng/mL, at all of the measured points. After 2 week of UVB irradiation, the VDd with 100 μω/cm^2^ group had its serum 25(OH)D levels dramatically improved to normal, and maintained this level at 3 week with no significant difference when compared with the normal diet group. The VDd with 50 μω/cm^2^ UVB irradiation group needed an additional 1 week to recover its 25(OH)D level to normal. The ceiling of 25(OH)D level was approximately 20 ng/mL at 3 week in the VDd with 25 μω/cm^2^ UVB irradiation group. Finally, in the VDd with 12.5 μω/cm^2^ UVB irradiation group, which was the lowest exposure, its 25(OH)D levels were never higher than 15 ng/mL, and maintained a level of approximately 10 ng/ml for the remainder of the measured points after 2 week. In general, the time effects of UVB irradiation on 25(OH)D level exhibited a significant time-trend improvement concerning the mean value. The ceiling of 25(OH)D occurred at 2 or 3 week after the start of exposure, and this level persisted afterwards. At 10 weeks-of-age, a decrease of 25(OH)D levels was observed among all irradiation groups, but seemed to be unrelated to receiving a VDd diet or UVB irradiation.

**Table 1:** Serum 25-hydroxyvitamin D level simulated by different doses of 308 nm UVB irradiation to VDd mice.

Group	Irradiation duration, week (N = 6 at each time point)					
	0	1	2^#^	3^#,$^	4^#^	5^&^
CA^b,c,d,e^	29.6 ± 3.6	26.2 ± 4.0	31.6 ± 4.4	37.0 ± 2.6	36.0 ± 4.7	24.6 ± 6.4
CB^a,c,d,e,f^	UD	3.3 ± 0.6	4.6 ± 2.0	3.2 ± 0.5	3.0 ± 0.0	4.3 ± 0.9
**VDd with UVB irradiation, μω/cm**^**2**^						
UVB 1, 12.5^a,b,d,e,f^	UD	4.7 ± 2.1	10.7 ± 2.2	10.2 ± 2.0	9.7 ± 3.1	9.7 ± 0.9
UVB 2, 25^a,b,c,e,f^	UD	8.8 ± 2.1	17.3 ± 4.2	21.1 ± 5.4	21.4 ± 8.5	19.0 ± 3.3
UVB 3, 50^a,b,c,d,f^	UD	16.6 ± 3.5	25.9 ± 5.6	35.1 ± 4.1	–	–
UVB 4, 100^b,c,d,e^	UD	26.3 ± 3.1	35.4 ± 4.7	39.9 ± 2.5	–	–

Data represented as mean ± SD. Unit: ng/mL.

The 25OHD levels lower than the detection limit of 3 ng/mL by Elecsys cobas e411 analyzer were marked as UD.

Unbalanced ANOVA with a two-way design was applied to inspect the effects of UVB dose per time and irradiation duration on 25-hydroxyvitamin D level. Differences of means between groups of UVB dose were tested by the Tukey–Kramer approach to adjust for multiple comparisons.

VDd, vitamin D deficiency; UVB, ultraviolet B; CA, control A group feeding with normal diet; CB, control B group feeding with VDd diet; UD, undetectable.

^a^*P* < 0.05 when compared with the mean of 25 (OH)D level in the CA group.

^b^*P* < 0.05 when compared with the mean of 25 (OH)D level in the CB group.

^c^*P* < 0.05 when compared with the mean of 25 (OH)D level in the UVB 1 group.

^d^*P* < 0.05 when compared with the mean of 25 (OH)D level in the UVB 2 group.

^e^*P* < 0.05 when compared with the mean of 25 (OH)D level in the UVB 3 group.

^f^*P* < 0.05 when compared with the mean of 25 (OH)D level in the UVB 4 group.

^#^*P* < 0.05 when compared with the mean of 25 (OH)D level at week 1.

^$^*P* < 0.05 when compared with the mean of 25 (OH)D level at week 2.

^&^*P* < 0.05 when compared with the mean of 25 (OH)D level at week 3.

### UVB irradiation time and serum 25(OH)D level

**Figure 1:**
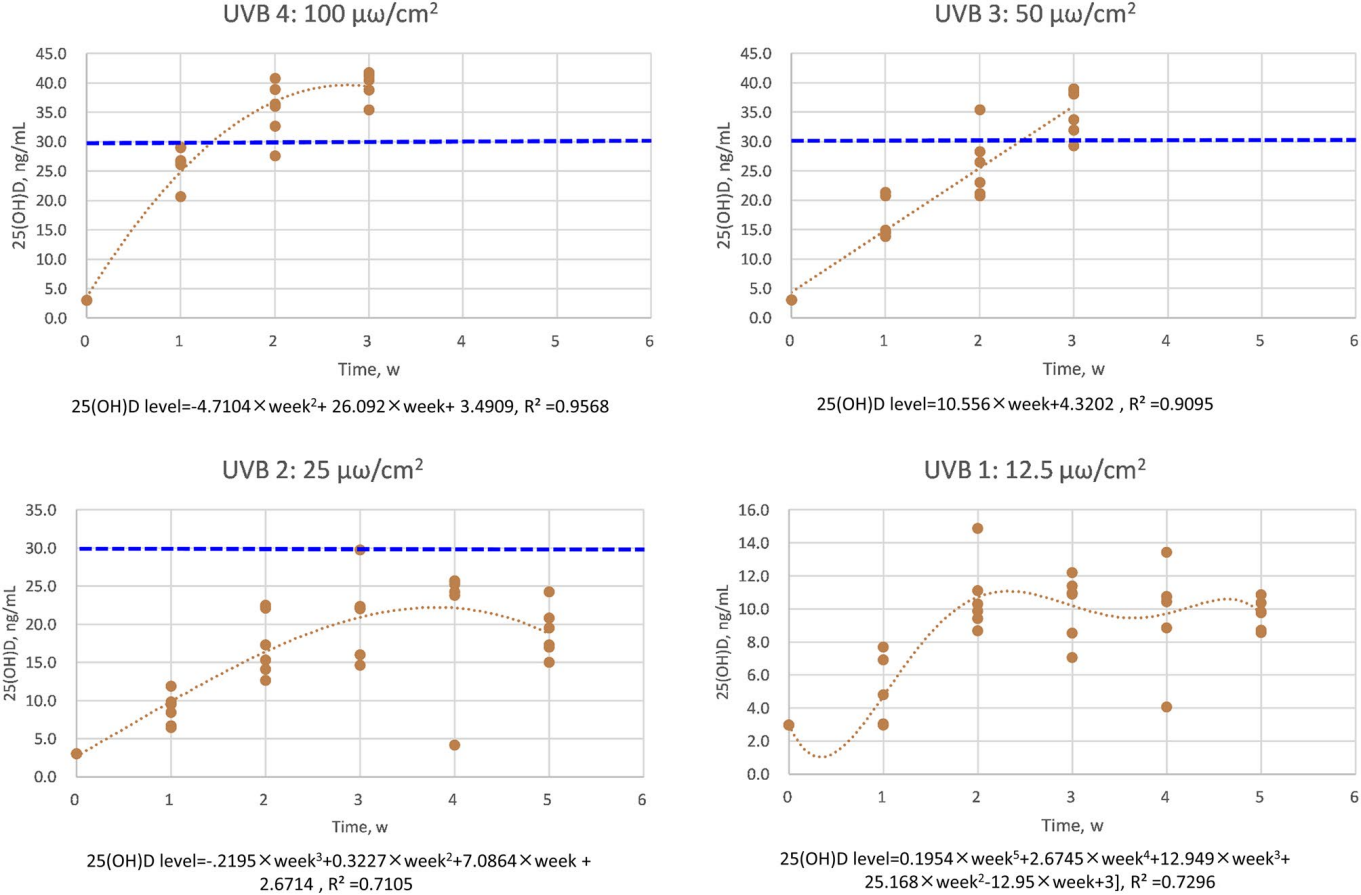
The distibution of 25(OH)D levels after 308 nm UVB irradiation in different exposure times according to dose. The optimal mathematical function to describe the associations between irradiation time and 25(OH)D level by different UBV irradiation dose was determined by maximal R square value.

Figure 1 presents the distribution of 25(OH)D levels after 308 nm UVB irradiation in different exposure times by various exposure doses (Fig. 1). Every dose exposure used in this study seemed to reach its own maximal level, followed by a plateau phase. The prediction relationship was established using a general linear model with polynomials of degrees of exposure time in the groups of UVB irradiation with 50 and 100 μω/cm^2^. A linear relationship was found in the group of VDd with 50 μω/cm^2^ UVB irradiation. In contrast, a quadratic and cubic curve relationship was found in the group of VDd with 100 and 25 μω/cm^2^ UVB irradiation, respectively. A more complex curve relationship was identified in the group of VDd with 12.5 μω/cm^2^. The prediction formulas were as follows:

$$\begin{aligned} & 100\;\mu \omega {\text{/cm}}^{2} :\;[25\left( {{\text{OH}}} \right)\;{\text{D}}\;{\text{level}} = - 4.7104 \times {\text{week}}^{2} + 26.092 \times {\text{week}} + 3.4909],\;{\text{R}}^{2} = 0.9568 \\ & 50\;\mu \omega {\text{/cm}}^{2} :\;[25\left( {{\text{OH}}} \right)\;{\text{D}}\;{\text{level}} = 10.556 \times {\text{week}} + 4.3202],\;{\text{R}}^{2} = 0.9095 \\ & 25\;\mu \omega {\text{/cm}}^{2} :\;[25\left( {{\text{OH}}} \right)\;{\text{D}}\;{\text{level}} = - .2195 \times {\text{week}}^{3} + 0.3227 \times {\text{week}}^{2} + 7.0864 \times {\text{week}} + 2.6714],\;{\text{R}}^{{2}} = 0.7105 \\ & 12.5\;\mu \omega {\text{/cm}}^{2} :\;[25\left( {{\text{OH}}} \right)\;{\text{D}}\;{\text{level}} = 0.1954 \times {\text{week}}^{5} + 2.6745 \times {\text{week}}^{4} + 12.949 \times {\text{week}}^{3} + 25.168 \times {\text{week}}^{2} - 12.95 \times {\text{week}} + 3],\;{\text{R}}^{{2}} = 0.7296 \\ \end{aligned}$$

### Cumulative doses of 308 nm UVB irradiation and serum 25(OH)D levels

**Figure 2:**
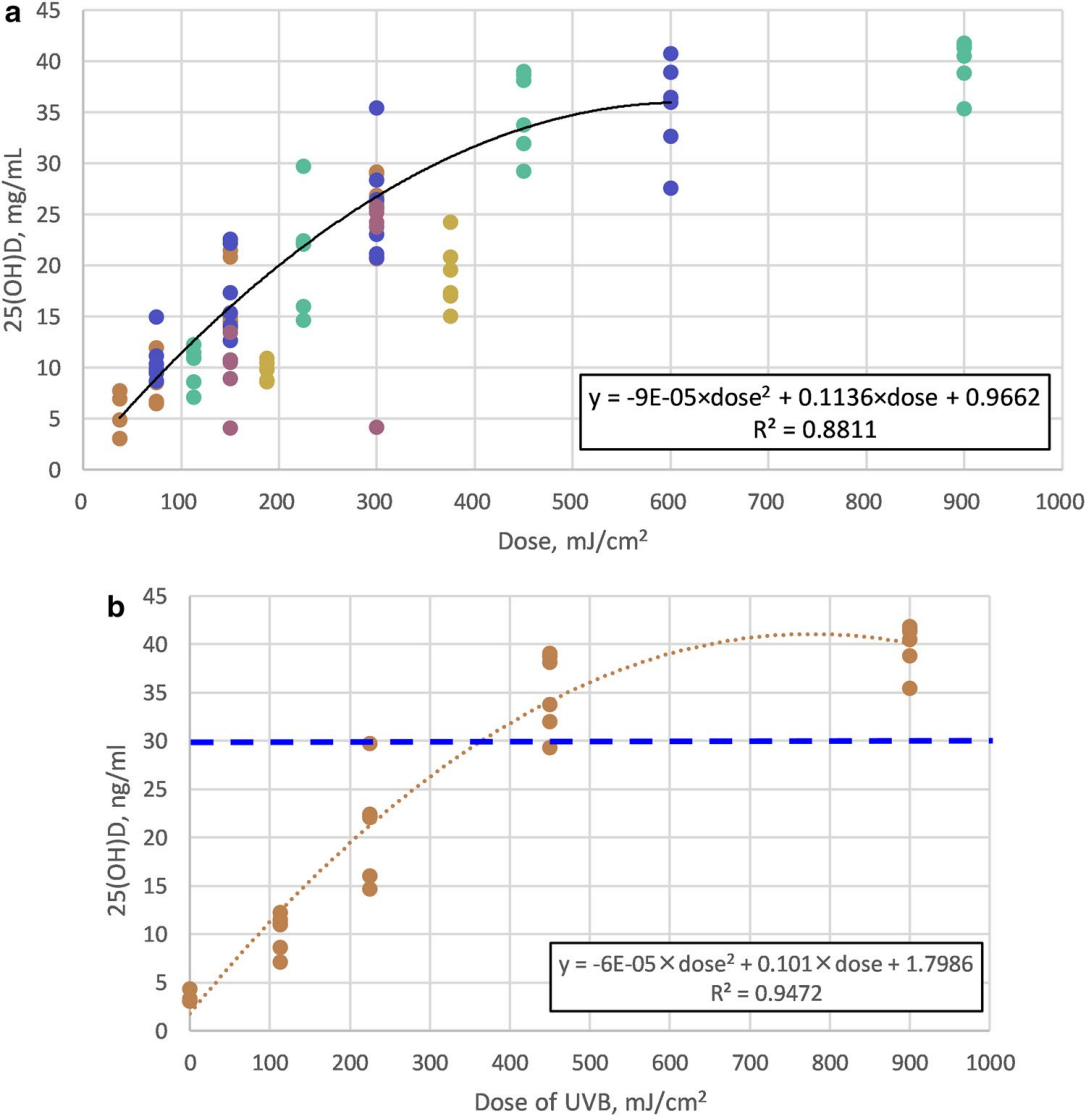
The distibution of 25(OH)D level and relationship with cumulative dosage of UVB irradiation. (**a**) For all time-point irradiation; (**b**) at 3 w irradiation. The optimal mathematical function to describe the associations between UBV irradiation dosages and 25(OH)D level at 3 w was determined by maximal R square value. The optimal dosage of UVB (355 mj/cm^2^) to reach 25(OH)D level at 30 ng/mL was estimated based on the mathematical function.

We created a scatter plot (Fig. 2a) to illustrate the relationship of 25(OH)D levels with cummulative doses from each UVB irradiation group. Based on this distribution, an almost linear pattern was demonstrated with those groups at and before 3 week of UVB exposure and 25(OH)D level below 40 ng/ml. The aformentioned relationship could be expressed as 25(OH)D level =  − 9E-05 × dose^2^ + 0.1136 × dose + 0.9662, R^2^ = 0.8811. Based on this mathemtical function, the estimated, optimal dose of 308 nm UVB for VDd mice to achieve 25(OH)D level at 30 ng/mL within 3 week irradiation was 355 mJ/cm^2^. However, if the irradiation was given for a duration longer than 3 week, such as our group of 25 μω/cm^2^ at 5 week with a total of 375 mJ/cm^2^, the relationship was not maintained.

### Cumulative doses of 308 nm UVB irradiation at 3 week and serum 25(OH)D levels

25(OH)D levels from each UVB irradiation group at 3 week were extracted and formed the following quadratic relationship: [25(OH)D level =  − 6E-05 × dose^2^ + 0.101 × dose + 1.7986, R^2^ = 0.9472] (Fig. 2b). Based on this mathemtical function, the estimated, optimal dose of 308 nm UVB for VDd mice to achieve 25(OH)D level at 30 ng/mL for 3 week irradiation was 355 mJ/cm^2^.

## Discussion

In this study, the effects of low-dose and narrow-band UVB at 308 nm on vitamin D production were precisely quantified in a mouse model. The various low-dose irradiations all increased 25(OH)D level and reached a plateau at no more than 3 week after UVB exposure. Importantly, the UVB dose at 50 μω/cm^2^ and higher could normalize severe VDd. We further identified the time-response curves for 25(OH)D synthesis, and the UVB dose of 50 μω/cm^2^ had a linear effect on improving VDd, while the remaining doses followed more complicated rules. An accumulated dosage of 355 mJ/cm^2^ UVB irradiation is estimated to normalize 25(OH)D level at approximately 30 ng/mL from severe VDd at 3 week.

Several studies used either low-dose or/and narrow-band UVB for VDd improvement^23,24,29,30^; but ours is the first one to demonstrate, with the center of wavelength at 308 nm, could normalize severe VDd. Narrow band UVB, first utilized as phototherapy for psoriasis, was previously shown, just as sunlight and broad band UVB, to be able to increase 25(OH)D. Since then, several studies investigated the effect of different wavelengths of UVB on VDd improvement, and it is now clear that wavelength affects cutaneous vitamin D synthesis in a hill distribution with the peak efficiency at 300 nm over the UVB wavelength spectrum^27,28^. Veronikis A.J. et al. in their recent study discovered that UVB-LED with the wavelength of 295 nm and irradiation of 117, 234, and 468 J/m^2^ were effective at generating vitamin D3 in human skin in vitro with a dose-response^23^. Our results further proved, even with less biological effect on vitamin D synthesis, UVB 308 nm of lower irradiation less than 1 SED (100 J/m^2^) can normalize the VDd. UVB at 300 nm, however, also produces the most intense deleterious effects on DNA, sunburn, and carcinogenesis. As a consequence, in an attempt to achieve an optimal balance, we chose the UVB wavelength at 308 nm, which had not been previously tested. Indeed, this band possesses several characteristics that are especially suitable for safety concerns when exposed chronically, including the following: less intensity of UVB adverse effects; part of natural sunlight reaching the Earth; and acceptable, although not the highest, efficacy on cutaneous vitamin D synthesis. We successfully increased, and even normalized, 25(OH)D level of VDd mouse using 308 nm UVB with low irradiance, even though other literature reported that wavelengths at 305 and 316 nm were much less efficient on cutaneous vitamin D synthesis^24,29^. Further investigations are requisite to confirm that long-term exposure to this UVB band would not cause adverse complications under low dosage.

This study is also the first study to measure and adjust irradiance to the preset target prior to each exposure to elucidate the effects of various low dosages of narrow-band UVB irradiation. Few existing studies measured irradiance before exposure, or used devices that could be adjusted to meet the preset target^31–35^. In addition, although many investigations have focused on the effects of UV irradiation dosage on vitamin D levels in vivo, some of them used irradiation that was much higher than SED, which makes further clinical applications as a “health lamp” less pragmatic^28,36^. Moreover, those studies that explored low-dose UVB, lower than SED and either broad or narrowband, shared the same problem that irradiance was estimated rather than measured^37,38^. This made information collected, once applied to health lamp development, less precise and inferable. Our results, in contrast, were robust and supported that the irradiance of 50 μω/cm^2^ or total accumulated irradiation of 355 mJ/cm^2^ within 3 week was sufficient to normalize severe VDd status to above 30 ng/ml. Indeed, even irradiance as low as 12.5 μω/cm^2^, which was the smallest irradiance in this study, could increase 25(OH)D by nearly 10 ng/ml. This effect could equal a daily oral supplement of 11.4–43.5 μg (≈500–1500 IU) vitamin D, which is beneficial for mild VDd^39^. It is worth noting that our very low baseline 25(OH)D level may constitute another reason why UVB exposure with such a low dose, i.e., only 0.28 of a SED (or 2.8 mJ/cm^2^ of erythemally effective UV radiation), could improve VDd^40,41^.

We further created mathematical functions for understanding the relationship between 25(OH)D level and different UVB dosages, and determining optimal overall UVB dosages in a mice model. We confirmed that serum 25(OH)D levels exhibited a linear pattern with UVB dosage and nonlinear pattern with exposure duration. Several studies have demonstrated a linear trend between 25(OH)D levels and UVB dosage^28,37,42,43^, but few of them examined the series change of 25(OH)D levels after UVB irradiation^37^. Indeed, the linear pattern with UVB dosage was so substantial that exposure duration had almost no effect on the model (Fig. 2a). This means that serum 25(OH)D levels after UVB irradiation of 1, 2, or 3 week were all predictable by the created formula based on the accumulated irradiation dosage, especially at the range before serum 25(OH)D recovered to the normal level at 30 ng/ml. This result indicated that the optimal dose of UVB irradiation for vitamin D synthesis under the experimental conditions may be approximately 40 μw/cm^2^ for 10 min and 5 days/week, at which time the total irradiation dosage equaled 355 mJ/cm^2^ (Fig. 2b). This overall 355 mJ/cm^2^ UVB dosage within 3 week to reach a sufficient vitamin D level can be further applied to explore how exposures with various frequencies would lead to the same result. The nonlinear pattern with exposure duration at various irradiances revealed that 3 week seemed requisite to reach the plateau. The role of exposure frequency is not clear concerning the duration of UVB irradiation necessary to achieve the plateau. In our study design, the frequency of five-time exposure per week led all of the groups to reach a plateau of their own 25(OH)D levels at 3 week. Other studies, however, suggested that a longer duration, even up to several months, was needed to reach a steady-state under different exposure frequencies^30^. We found that at least 3 week of exposure was needed before testing if 25(OH)D reached the target. Further study is required to identify the optimal frequency for health lamp development.

Our study possesses two main strengths. First, we developed an adjustable energy narrow-band UVB-light-emitting diode (LED) for animals with an irradiance detector, which can precisely quantify and test the effects of dose, time, frequency, and duration of UVB irradiation on vitamin D synthesis. To the best of our knowledge, the current study is the only investigation to precisely measure irradiance when delivering each UVB exposure. Furthermore, our LED light source with an adjustable dose function can achieve more stringent experimental conditions than previous studies through activating light intensity control and low ambient light interference. Second, we have established different time-response courses of vitamin D after different low-dose UVB exposures in a mouse model, providing valuable data to support future designs of optimal health lamps to improve or prevent VDd.

This study also had certain limitations worth noting. First, body surface area (BSA) is another determinant of increasing 25(OH)D level when exposed to UVB, which was roughly, but not precisely, controlled in this study. Further research is needed to identify the optimal BSA, especially so that the human face and hands can synthesize vitamin D more efficiently. Second, skin pigmentation constitutes another critical determinant that may affect the response to UVB, which is unable to be manipulated in the mouse model. Third, like most of the extant literature, we did not evaluate skin damage resultant from UVB irradiation, either in terms of sunburn or carcinogenesis. However, the optimal UVB dose for sufficient vitamin D synthesis that we found in the current study (between 25 and 50 μω/cm^2^ for 10 min, ~ 0.56–1.12 SED) was quite small when compared with not only the expected MED in subjects with skin types I to IV (150–600 J/m^2^) , but also the dose that we measured at an outdoor environment in northern Taiwan (24.782754°, 120.996617°) at 12:00 pm on September 01, 2015^27,44^.

## Conclusion

The present study demonstrated that narrow-band UVB 308 nm provided by a UV-LED device is effective in normalizing serum vitamin D from severe VDd at a dose of no more than 355 mJ/cm^2^ within 3 week. Moreover, total exposure irradiation is related to the highest 25(OH)D level, and how soon this level can be reached is most probably related to exposure frequency. This study provides important information for the future development of therapeutic UV-LED devices with less side effects for the treatment or prevention of vitamin D deficiency related disorders.

## Material and methods

### Mice and diet

Inbred C57BL/6 female mice at three weeks-of-age were purchased from the National Laboratory Animal Center (Taipei, Taiwan). The animals were randomly housed with four mice per cage, with a 12-h interval alternating of light and dark cycle in a humidity-controlled room. The normal diet group was fed with Altromincatalogue n0.1310 (Altromin International, Lage, Germany) during the experimental period. For the VDd group, the mice were fed with a VDd diet manufactured by Dyets, Inc. (Bethlehem, PA, U.S.A.) for four weeks prior to the study and lasted until the end of the experiment. The durations of VDd diet feeding before being ready for the experiment were determined based on a previous study^38^, and the results of our preliminary results at 4, 6, and 8 week, which was 4 week in this study. The seven-week-old VDd mice further received different doses of narrow-band UVB irradiation, as indicated. All experimental protocols used in this study were approved by the Institutional Animal Care and Use Committee of Kaohsiung Medical University (no: 105181) and carried out in compliance with the ARRIVE guidelines. These procedures were also performed in strict accordance with Taiwanese law on the care and usage of laboratory animals.

### Narrow-band UVB exposure system development

The UVB exposure system for mice used in this study was developed by the Taiwan Instrument Research Institute, National Applied Research Laboratories. The system comprises a UV-LED array, cooling module, power supplier, cylindrical darkroom, and a measurement device for irradiance determination, as shown in Supplementary figure S1a online. The LED light source module is presented in Supplementary figure S1b online. Nine UV-LED chips are evenly arranged within a 3 × 3 cm^2^ area on the metal substrate. We determined the wavelength of the LED based on the findings of a previous review^28^, as well as our own solar spectrum measurements and inferences^27^, which suggested that a light source within the 305–315 nm band should be the optimal wavelength to balance vitamin D synthesis efficiency and UVB adverse effects, such as sunburn and carcinogenesis. Therefore, we chose a 308 nm LED for this animal study. The light source module has an adjustable function of brightness, and the instability of the optical output power is less than 2% of the set value. The operator would manually adjust the light intensity, and the smallest adjustment level is 1/100 of the highest irradiance (~ 120 μω/cm^2^). The light source is mounted on a cylindrical darkroom module. The darkroom is fabricated by 3D printing with black plastic material to accommodate mice in a space of 85 cm^2^ for UVB exposure. In order to stabilize light emission of the LED, a large heat dissipation fin is utilized for cooling. The interior view of the darkroom is shown in Supplementary figure S1b online. The light beam emitted by the LED array illuminates the bottom of the darkroom with sufficient height to make the illumination uniformity greater than 95%. A replaceable module equipped with an optical sensor (Thorlabs S120VC) was also utilized to confirm the irradiance to which the mice were exposed during the experiment. The irradiance system was calibrated according to the International Organization for Standardization17025, the International Electrotechnical Commission Guide 115, and JCGM100:2008 (GUM) protocol procedures to ensure traceability of study results (Supplementary Fig. S2a and S2b online). The irradiance in the darkroom was recorded prior to the mice being sent into the darkroom for UVB exposure, which enabled us to ensure that the mice were exposed to the target dose each time. Under this experimental design, irradiation conditions were precisely controlled.

### Experimental design and serum 25(OH)D levels measurement

**Figure 3:**
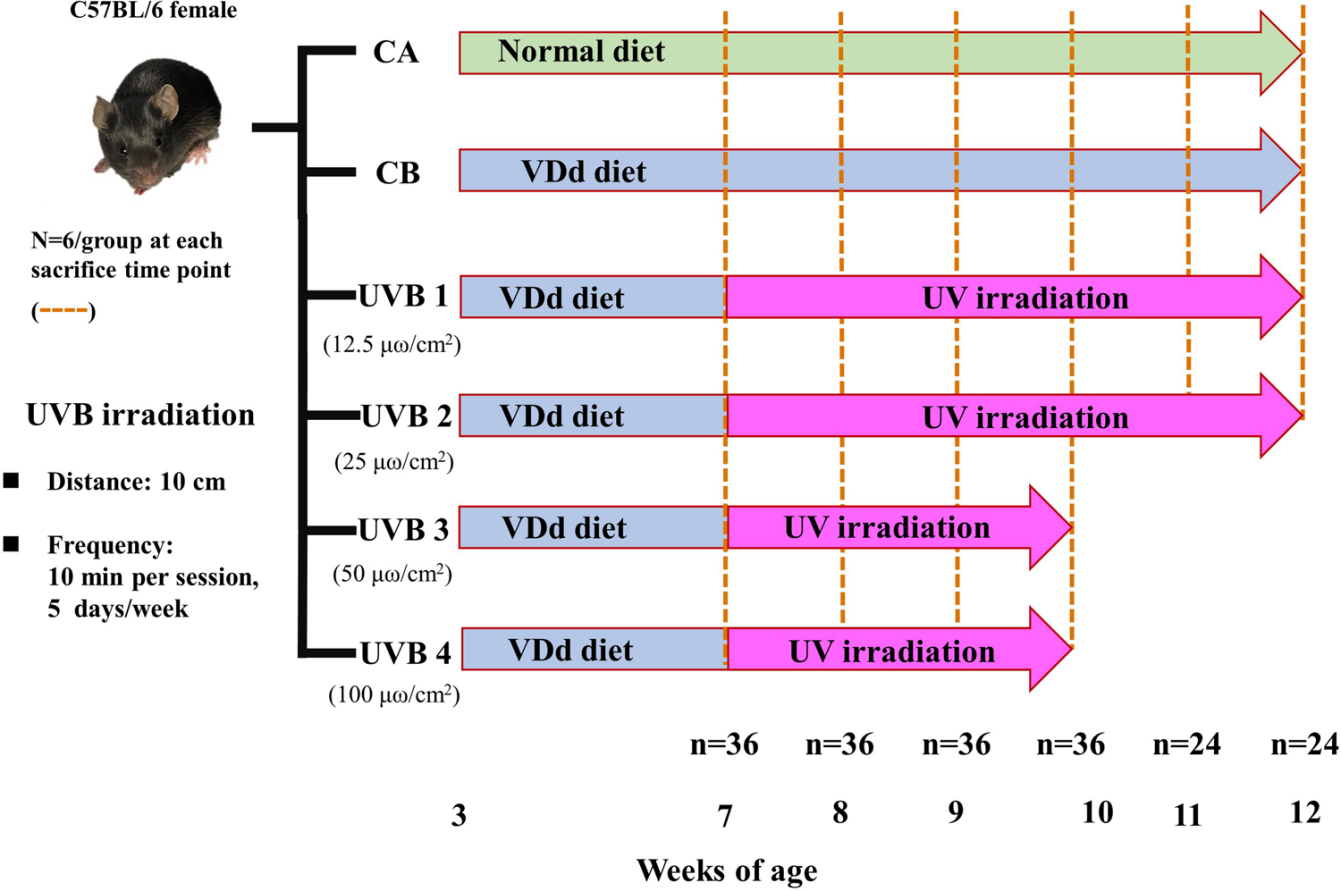
The experimental protocol. Inbred C57BL/6 female mice at three-weeks-old were randomly divided into the following six groups: (1) Control A (CA), fed a normal diet during the entire experiment; (2) Control B (CB), fed a vitamin-D deficient (VDd) diet during the entire experiment; (3) UVB 1, fed a VDd diet during the entire experiment and exposed to 12.5 μω/cm^2^ UVB after being fed the VDd diet for 4 weeks; (4) UVB 2, fed a VDd diet during the entire experiment and exposed to 25 μω/cm^2^ UVB after being fed the VDd diet for 4 weeks; (5) UVB 3, fed the VDd diet during the entire experiment and exposed to 50 μω/cm^2^ UVB after being fed the VDd diet for 4 weeks; and (6) UVB 4, fed the VDd diet during the entire experiment and exposed to 100 μω/cm^2^ UVB after being fed the VDd diet for 4 weeks. For all six groups, the same sacrifice time points were at 7, 8, 9, and 10 weeks-of-age; and for the groups of CA, CB, UVB 1 and UVB 2, there were two additional points at 11 and 12 weeks-of-age. The frequency of UVB irradiation was 10 min /day and 5 days/week.

The mice at three weeks-of-age (n = 192) were divided into six groups, including the regular diet group (the CA group), and VDd groups with and without irradiation (the CB group) (Fig. 3). We further divided the VDd with irradiation group (the UVB 1- 4 groups) to receive four different irradiation doses of UVB, which were 12.5, 25, 50, and 100 μω/cm^2^, respectively. The dorsal region of the mice (~ the 35% of total skin area of the mouse) were shaved using electric clippers 1 day before UVB irradiation. The distance of UVB irradiation was set at 10 cm, with a duration of 10 min per session and 5 days/week. The two VDd groups exposed to higher doses, 50 and 100 μω/cm^2^, received irradiation for 3 weeks since our pilot study demonstrated that this duration was adequate to normalize severe VDd. The remaining two groups exposed to extremely low doses, 12.5 and 25 μω/cm^2^, received irradiation for 5 week. The total mice were crosssectionally sacrificed at 0 (n = 36), 1 (n = 36), 2 (n = 36), 3 (n = 36), 4 (n = 24), and 5 (n = 24) week after UVB irradiation, as indicated.

After completing the UBV irradiation, the mice were sacrificed by CO_2_ asphyxiation with flow rate of 5 L/min. Once death was confirmed, blood samples were immediately collected from mice heart chambers using a 15 mL centrifuge tube. Serum was obtained by centrifuging at 3,000 rpm for 10 min in a refrigerated centrifuge at 4 ℃. The specimen was then stored in a refrigerator at – 80 ℃. Most of the serum samples were analyzed by batch within 1 week post-collection, and no later than 3 week post-collection. The concentrations of plasma total 25(OH)D were analyzed using a Elecsys cobas e411 analyzer (Elecsys Vitamin D total; Roche Diagnostics, Switzerland, No. 25596501) with the detection limit of 3 ng/mL. The inter-assay and intra-assay coefficient of variability for 25(OH)D meansurments were 4.00% and 3.47%, respectively.

### Statistical analysis

All of the 25(OH)D concentration in each group was exhibited as mean ± standard deviation in ng/mL. To explore the optimal mathematical function in describing relationships between UVB irradiation time and 25(OH)D level for each irradiation group and determining the optimal cut-off value of UVB dosage for normalizing vitamin D level, we applied a general linear model with polynomials of degrees of exposure time from 1 to 5 weeks. The relationships between 25(OH)D level and UVB dosages within and at 3 week were determined by maximal R square value. Unbalanced ANOVA with a two-way design was applied to inspect the effects of UVB dose per time and irradiation duration on 25(OH)D level. Differences of means between different groups were tested by the Tukey–Kramer approach to adjust for multiple comparisons. Statistical analyses and figures were carried out using SAS 9.4 software (SAS Institute Inc., Cary, NC, U.S.A.) or Windows Microsoft PowerPoint or Excel 2016, if appropriate. A two-tailed *P* < 0.05 indicated statistical significance.

## Supplementary Information

Supplementary Information.

Supplementary figure 1.

Supplementary figure 2.

Supplementary figure 3.

Supplementary figure 4.

## Abbreviations

25(OH)D: 25-Hydroxyvitamin d
BSA: Body surface area
LED: Light-emitting diode
SED: Standard erythema dose
UVB: Ultraviolet B
VDd: Vitamin D deficiency
