# Supplementary material for: Low dose ultraviolet B irradiation at 308 nm with light-emitting diode device effectively increases serum levels of 25(OH)D
Source: Sci Rep. 2021 Jan 28;11:2583. doi: 10.1038/s41598-021-82216-1 (PMC7844009; doi:10.1038/s41598-021-82216-1)
Supplement: Supplementary file 1 — Supplementary Information. [file 41598_2021_82216_MOESM1_ESM.docx]

**Low dose Ultraviolet B Irradiation at 308 nm with Light-emitting Diode Device Effectively Increases Serum Levels of 25(OH)D**

Ming-Yen Lin Ph.D.^a,b#^, Lee Moay Lim M.D.^a,g#^ , Siao-Ping Tsai M.S.^a^, Feng-Xuan Jian M.S.^a^, Shang-Jyh Hwang M.D.^a,b, c,d^, Yu-Hsuan Lin Ph.D.^e*^, and Yi-Wen Chiu M.D.^a,b*^

^a^Division of Nephrology, Department of Internal Medicine, Kaohsiung Medical University Hospital, Kaohsiung Medical University, Kaohsiung 807, Taiwan

^b^Department of Renal Care, College of Medicine, Kaohsiung Medical University, Kaohsiung 807, Taiwan

^c^Graduate Institute of Medicine, College of Medicine, Kaohsiung Medical University, Kaohsiung 807, Taiwan

^d^Institute of Population Health Sciences, National Health Research Institutes, Miaoli 350, Taiwan

^f^Taiwan Instrument Research Institute, National Applied Research Laboratories, Hsinchu 300, Taiwan

^g^Faculty of Medicine, College of Medicine, Kaohsiung Medical University, Kaohsiung 807, Taiwan

^#^The authors are equal contributors

^＊^Corresponding authors

Yi-Wen Chiu, M.D., email: chiuyiwen@gmail.com

Yu-Hsuan Lin, Ph.D., email: marklin@itrc.narl.org.tw

No. 100, TzYou 1^st^ Rd.

Sanmin District, Kaohsiung City 80708, Taiwan

Tel.: 886 7 3121101-7351-11; Fax: 886 7 3122810

**Short running title:** Low-dose narrow-band UVB and 25(OH)D level

**Abbreviations:** 25-hydroxyvitamin d, 25(OH)D; body surface area, BSA; light-emitting diode, LED; standard erythema dose SED; ultraviolet B, UVB; vitamin D deficiency, VDd.

**Supplemental figure legend**

**Fig. S1.** The developed 308 nm ultraviolet B (UVB) irradiation system.

**S1a.** The system consists of a UV-LED array, cooling module, power supplier, cylindrical darkroom, and a measurement device.

**S1b.** Functional diagram of the developed UVB exposure system. The replaceable module is the location to insert the power meter as an optical sensor. We designed the replaceable module to be at a height that was almost equal to the mouse, which enabled each exposure dose to be closely monitored and controlled.

**Fig. S2.** The intensity for a spectrum of the 308 nm ultraviolet B (UVB) irradiation system with 12.5, 25, 50, 100, and 300 μω/cm^2^.

**S2a.** The measured intensity for a spectrum of the 308 nm ultraviolet B (UVB) irradiation system with 12.5, 25, 50, 100, and 300 μω/cm^2^.The absolute spectrum and absolute optical intensity of the UV LED source were measured with a calibrated system. The system consists of a standard light source (DH-3, Ocean Optics), spectrometer (USB2000+, Ocean Optics), and precise optomechanical components. The light source was calibrated according to the International Organization for Standardization 17025, the International Electrotechnical Commission Guide 115, and JCGM100:2008 (GUM) protocol procedures to ensure traceability of study results.

**S2b.** The erythemal weighted intensity for a spectrum of the 308 nm ultraviolet B (UVB) irradiation system with 12.5, 25, 50, 100, and 300 μω/cm^2^. Erythemal weights for different wavelengths were obtained from updated formulas defined by the International Commission on Illumination (CIE)(1).

**Reference**

1. Schmalwieser AW, Wallisch S, Diffey BJP, Sciences P. A library of action spectra for erythema and pigmentation. 2012;11(2):251-68.
