# Supplementary figures and images for: Low dose ultraviolet B irradiation at 308 nm with light-emitting diode device effectively increases serum levels of 25(OH)D
Source: Sci Rep. 2021 Jan 28;11:2583. doi: 10.1038/s41598-021-82216-1 (PMC7844009; doi:10.1038/s41598-021-82216-1)

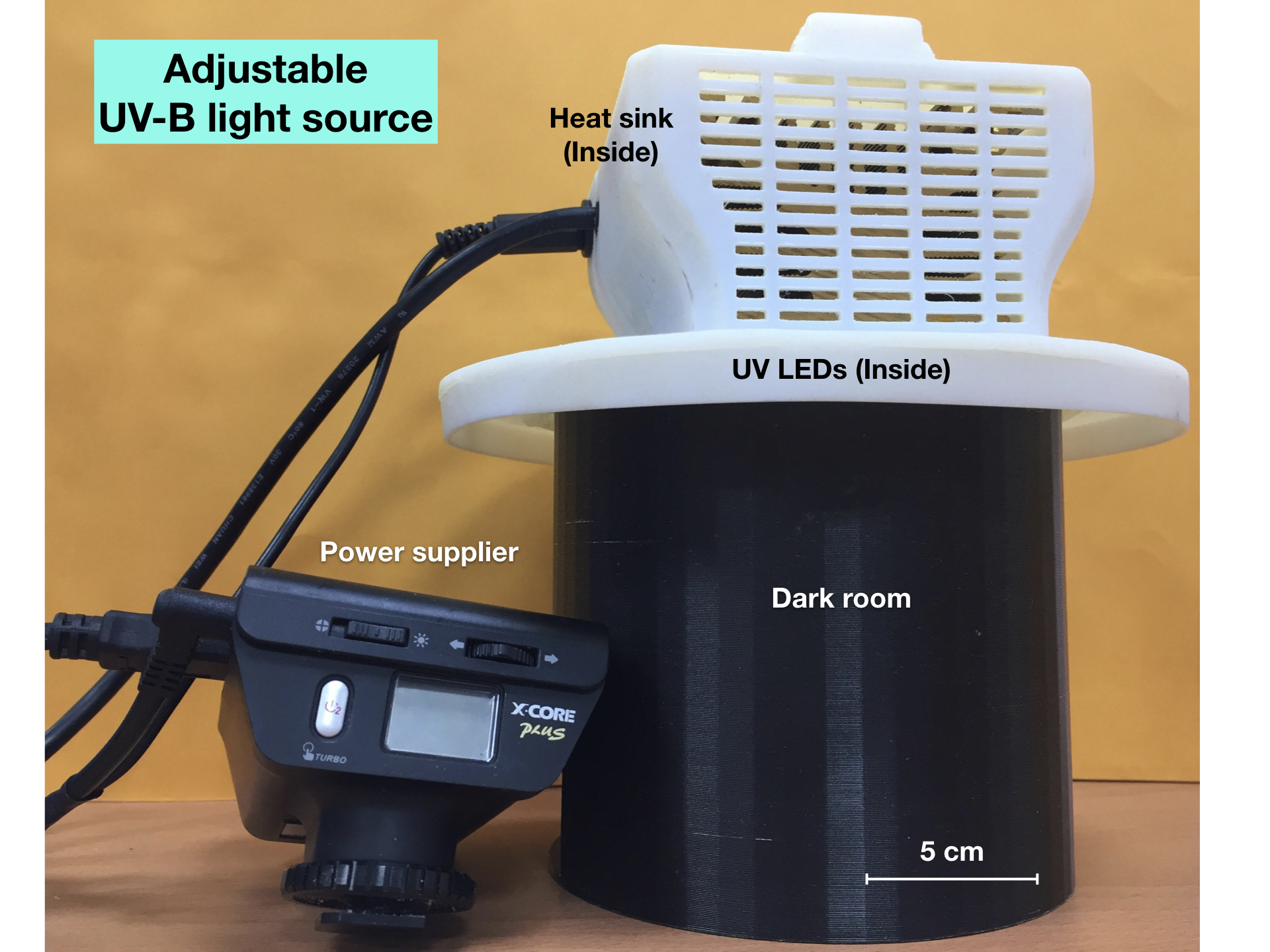

Supplement: Supplementary file 2 — Supplementary figure 1. [file 41598_2021_82216_MOESM2_ESM.jpeg]

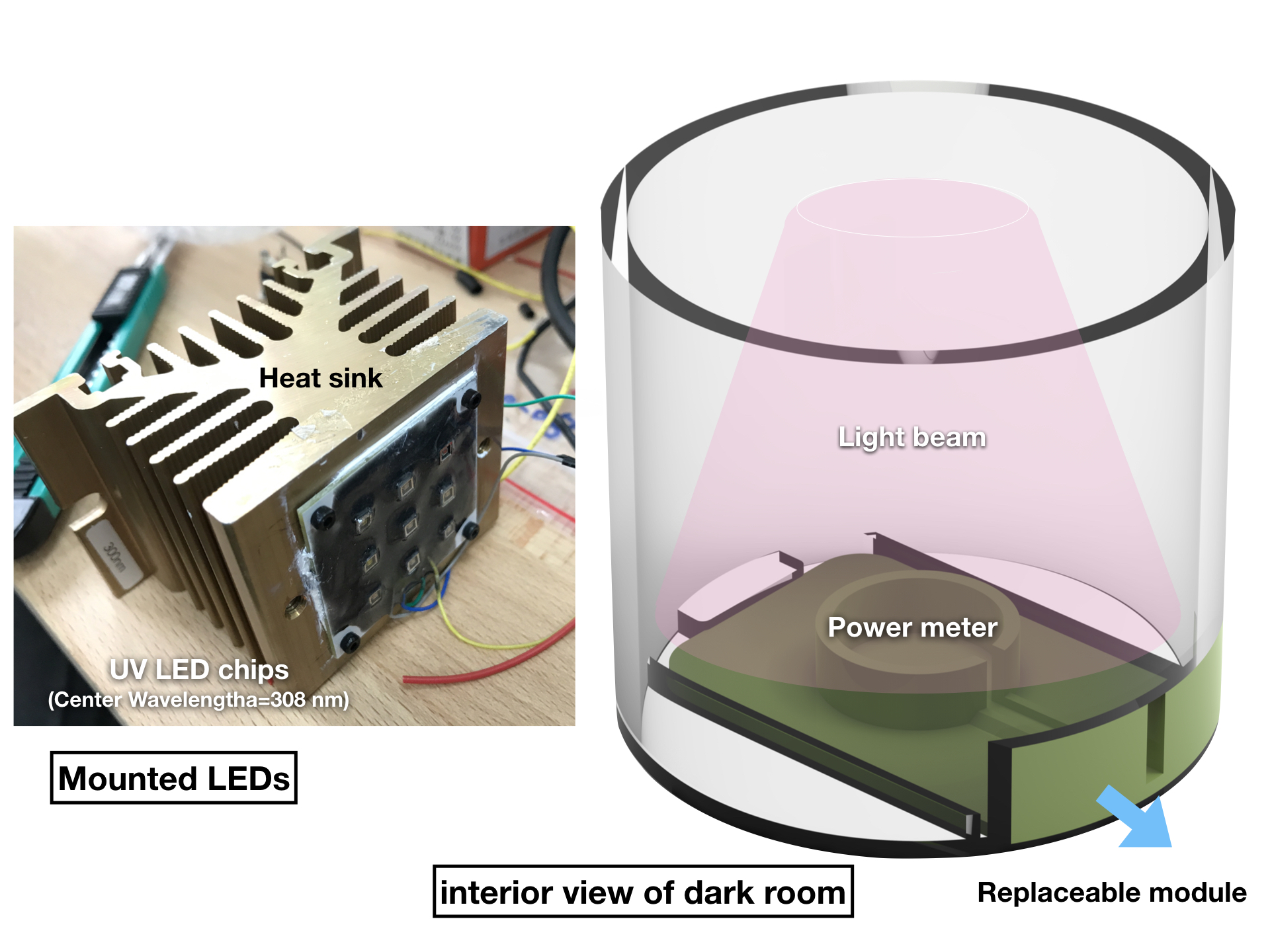

Supplement: Supplementary file 3 — Supplementary figure 2. [file 41598_2021_82216_MOESM3_ESM.jpeg]

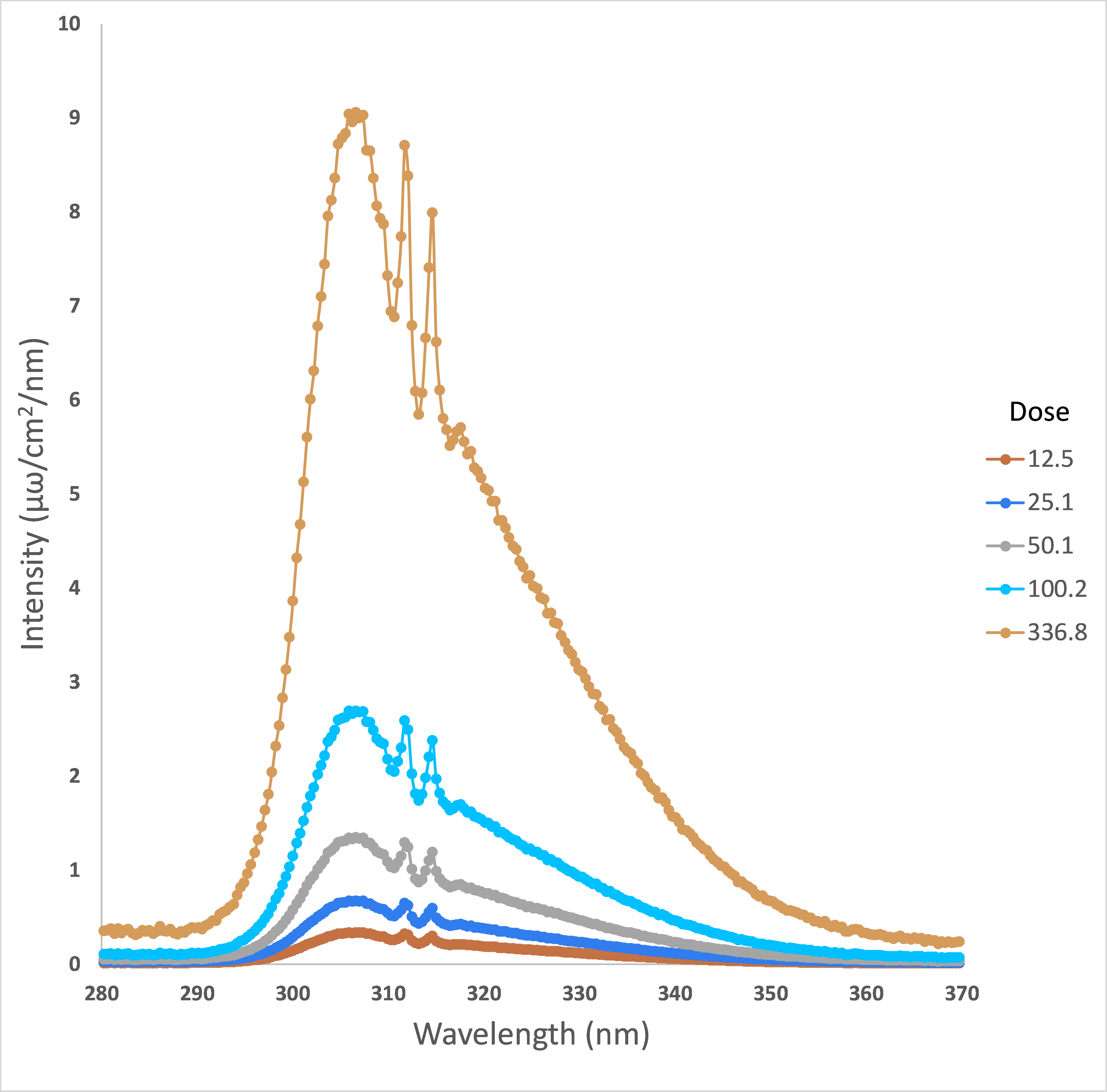

Supplement: Supplementary file 4 — Supplementary figure 3. [file 41598_2021_82216_MOESM4_ESM.tif]

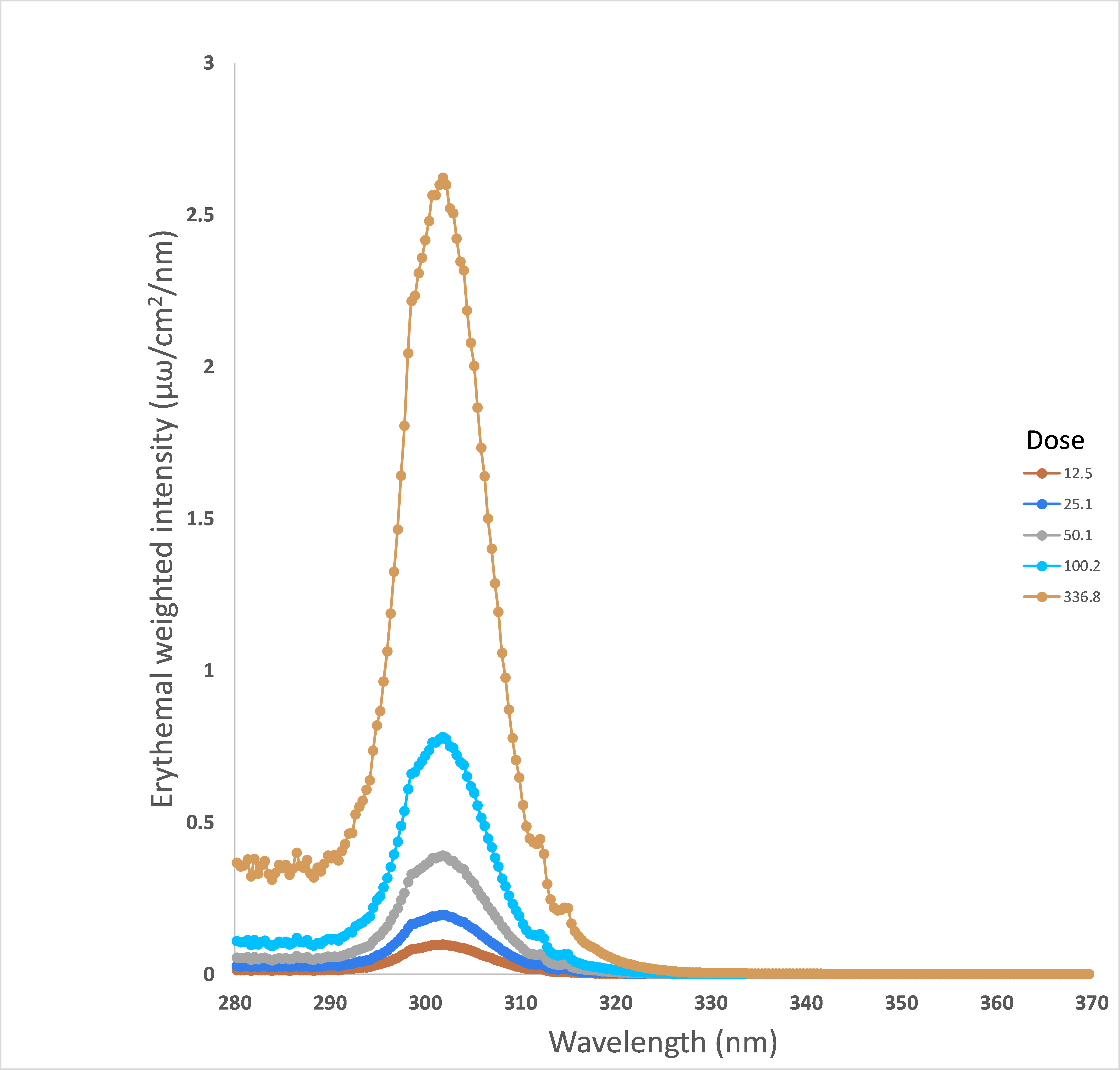

Supplement: Supplementary file 5 — Supplementary figure 4. [file 41598_2021_82216_MOESM5_ESM.tif]
